# Crusted scabies; a 2-year prospective study from the Northern Territory of Australia

**DOI:** 10.1371/journal.pntd.0008994

**Published:** 2020-12-18

**Authors:** Tasnim Hasan, Victoria L. Krause, Christian James, Bart J. Currie

**Affiliations:** 1 Centre for Disease Control, Northern Territory Top End Health Services, Darwin, Northern Territory, Australia; 2 Menzies School of Health Research, Charles Darwin University, Darwin, Northern Territory, Australia; 3 Royal Darwin Hospital and Northern Territory Medical Program, Darwin, Northern Territory, Australia; Michigan State University, UNITED STATES

## Abstract

**Background:**

Scabies is listed as a neglected tropical disease by the World Health Organization. Crusted scabies affects vulnerable and immunosuppressed individuals and is highly contagious because of the enormous number of *Sarcoptes scabiei* mites present in the hyperkeratotic skin. Undiagnosed and untreated crusted scabies cases can result in outbreaks of scabies in residential facilities and can also undermine the success of scabies mass drug administration programs.

**Methods and principal findings:**

Crusted scabies became a formally notifiable disease in the Northern Territory of Australia in 2016. We conducted a 2-year prospective study of crusted scabies cases notified between March 2016 and February 2018, with subsequent follow up for 22 months. Demographics, clinical and laboratory data, treatment and outcomes were analysed, with cases classified by severity of disease.

Over the 2-year study period, 80 patients had 92 episodes of crusted scabies; 35 (38%) were Grade 1 crusted scabies, 36 (39%) Grade 2 and 21 (23%) Grade 3. Median age was 47 years, 47 (59%) were female, 76 (95%) Indigenous Australians and 57 (71%) from remote Indigenous communities. Half the patients were diabetic and 18 (23%) were on dialysis for end-stage kidney failure. Thirteen (16%) patients had no comorbidities, and these were more likely to have Grade 3 disease. Eosinophilia was present in 60% and high immunoglobulin E in 94%. Bacteremia occurred in 11 episodes resulting in one fatality with methicillin-susceptible *Staphylococcus aureus* bacteremia. Two other deaths occurred during admission and 10 others died subsequent to discharge consequent to comorbidities. Treatment generally followed the recommended guidelines, with 3, 5 or 7 doses of oral ivermectin depending on the documented grade of crusted scabies, together with daily alternating topical scabicides and topical keratolytic cream. While response to this therapy was usually excellent, there were 33 episodes of recurrent crusted scabies with the majority attributed to new infection subsequent to return to a scabies-endemic community.

**Conclusions:**

Crusted scabies can be successfully treated with aggressive guideline-based therapy, but high mortality remains from underlying comorbidities. Reinfection on return to community is common while scabies remains endemic.

## Author summary

Undiagnosed crusted scabies patients can be index cases in outbreaks of scabies in residential facilities such as nursing homes and can undermine the success of scabies mass drug administration programs, but literature about this debilitating disease is very limited.

The study describes a large cohort of patients with crusted scabies who have been treated according to established local guidelines. While the success of the initial clinical response supports the guideline-based treatment regimen for crusted scabies, with only one patient dying with scabies-related sepsis, the subsequent mortality remains high, reflecting the large burden of comorbidities in the cohort. In addition, there was a high rate of recurrent crusted scabies, attributed to new infection with scabies once the cured patients return to their scabies-endemic communities. Crusted scabies can be successfully treated using robust guidelines including multiple doses of ivermectin together with topical therapy. However, community-based initiatives such as support for ‘scabies free zones’ are needed to prevent re-infection and recurrent disease in those individuals who are susceptible to developing crusted scabies. Undiagnosed crusted scabies is a threat to the success of scabies mass drug administration programs and will continue to drive scabies outbreaks in residential facilities.

## Introduction

Crusted scabies is a highly contagious dermatological infection with *Sarcoptes scabiei* mites. While scabies infection typically involves fewer than 15 mites, patients with crusted scabies can have millions of mites in the hyperkeratotic skin and scales that may involve much of the body surface. The immunopathogenetic basis of susceptibility that drives progression from ordinary to crusted scabies remains unclear, but associations include immunosuppressive therapy, infection with human immunodeficiency virus (HIV) and human T-lymphotropic virus-1 (HTLV-1) and those in aged-care facilities with stroke or other debilitating conditions [1,2]. While there are no published incidence rates for crusted scabies, the global burden of scabies has been estimated at 200–300 million cases a year [1] and population point prevalence up to 70% is documented [3].

The importance of diagnosing and treating crusted scabies is both for the individual, as mortality from secondary sepsis can be very high; and for the community, as unidentified cases of crusted scabies can be core transmitters to family members and the broader community. It has increasingly been recognised even in developed countries that outbreaks of scabies in residential aged-care facilities may be traced back to a previously unidentified index case with crusted scabies [2,4]. In 2016, crusted scabies was made a notifiable disease in the Northern Territory (NT) of Australia. This has enabled a study of demographic data, comorbidities, laboratory findings, treatment and outcomes for confirmed crusted scabies prospectively notified over a 2-year period.

## Methods

### Ethics statement

Ethical approval for this study was obtained from the Human Research Ethics Committee of the NT Department of Health and Menzies School of Health Research (HREC 2018–3163). As crusted scabies is a legislated notifiable disease in the NT, individual written consent was not obtained.

The NT makes up 15% of the Australian landmass but with a population of only 250,000. Approximately one third of the population are Indigenous Australians in comparison to 2·5% of the remaining Australian population [5]. Half of the NT Indigenous population live in rural or remote areas, often in small communities with substantial overcrowding, difficult to maintain health hardware and comparatively poor health outcomes [5]. Scabies is endemic in most of these communities [3].

The NT Health’s Centre for Disease Control (NT CDC) has a NT notifiable diseases system (NTNDS) which records all notifiable diseases fulfilling defined case definitions. We prospectively identified all cases of crusted scabies notified to the NT CDC over the 2 years from March 1, 2016 to February 28, 2018, documenting a primary outcome of mortality and a secondary outcome of recurrent crusted scabies over the subsequent 22 months until December 31, 2019. The sample size was therefore determined by the numbers of notified cases over the 24-month period. We used the NT case definition for crusted scabies which requires microbiological confirmation of presence of *Sarcoptes scabiei* mites from skin scrapings together with clinical skin changes (crusting and scaling) consistent with crusted scabies. The clinical skin assessment must be confirmed visually directly or from telehealth imaging by a specialist dermatologist or infectious diseases physician. Recurrent crusted scabies was defined as a notification fulfilling the case definition for crusted scabies which occurred after the planned treatment completion day for the prior episode of crusted scabies.

We classified the severity of crusted scabies using the formal grading scale (Grades 1–3) previously developed at Royal Darwin Hospital^6^ and now also used elsewhere (Box 1) [2]. Recommended therapy was as in the NT treatment guidelines for crusted scabies; dependent on the grading scale, three (Grade 1), five (Grade 2) or seven (Grade 3) doses of ivermectin are given, together with initially daily alternating keratolytic cream and either topical 25% benzyl benzoate +/- 5% tea tree oil or 5% permethrin cream (Box 1) [6–8]. We documented the actual therapy received for each patient, including noting the total number of doses of ivermectin. The NT guidelines for crusted scabies have been used internationally [2,9].

**Box 1. Categorising the grade of scabies and therapy**

A: Distribution of crusting
  1. Wrists, web spaces, feet (<10% TBSA)
  2. 1 plus forearm, lower legs, buttocks, trunk OR 10–30% TBSA
  3. 2 plus scalp OR >30% TBSA
B: Crusting/Shedding
  1. Mild crusting (<5mm depth), minimal skin shedding
  2. Moderate (5-10mm) crusting, moderate skin shedding
  3. Severe (>10mm), severe skin shedding
C: Past episodes
  1. No previous episodes
  2. 1–3 hospitalisations for crusted scabies OR depigmentation of elbow, knees
  3. > = 4 previous hospitalisations OR depigmentation as above AND legs/back, or residual skin thickening
D: Skin condition
  1. No cracking or pyoderma
  2. Multiple pustules or weeping sore or superficial skin cracking
  3. Deep skin cracking with bleeding, widespread exudates

Grade 1: Total score 4–6

Grade 2: Total score 7–9

Grade 3: Total score 10–12

Treatment

Grade 1: 3 doses of ivermectin, days 0,1,7

Grade 2: 5 doses of ivermectin, days 0,1,7,8,14

Grade 3: 7 doses of ivermectin, days 0,1,7,8,14,21,28

TBSA total body surface area

Adapted from Davis et al (2013) [6]

The NTNDS prospectively collects ‘core’ data on age, sex, Indigenous status, date of diagnosis, testing and geographical details. The major metropolitan areas of Darwin and Alice Springs were defined as urban centres, rural centres included Katherine and Tennant Creek and all other communities in the NT were considered remote. In addition, electronic and paper medical records were subsequently audited retrospectively to identify comorbidities including diabetes, end-stage kidney failure on dialysis, immunosuppression and hazardous alcohol use (60g alcohol average daily for males, and 40g alcohol average daily for females). Hospital length of stay, requirement for intensive care therapy and presence of secondary skin infections were documented. Treatment prescribed was recorded together with its completion according to the grade-based recommended number of doses of ivermectin and outcomes, including documentation of negative skin scrapings if performed. Laboratory data included: full blood count with neutrophil, lymphocyte and eosinophil count; total imunoglobulin E (IgE); C3 and C4; and serology for HIV and HTLV-1. Data were entered into Microsoft Excel 2016 and tabulated with simple descriptive statistics.

## Results

We identified 92 episodes of crusted scabies in 80 patients over the 24 months between March 1, 2016 and February 28, 2018. Two patients had 3 episodes of crusted scabies and 8 patients had 2 episodes within this two-year period. Time between 2 episodes for an individual varied between 1 and 18 months. Of the 92 episodes of crusted scabies, 35 (38%) were identified as Grade 1 crusted scabies, 36 (39%) were Grade 2 and 21 (23%) were Grade 3 crusted scabies. Demographic features and comorbidities for the 80 individuals are summarised in Table 1, with age and location taken from the most severe presentation with crusted scabies over the 2 years. Seventy-six of 80 (95%) patients identified as Indigenous Australians and 47 (59%) were female. There were only 2 children under the age of 16 years, one of whom had 2 episodes of crusted scabies over the 2 years. Both children were Indigenous with no identifiable comorbidities and they were aged 3 months, 4 years and 6 years at the time of each episode. Both lived in crowded houses in remote communities with poor health hardware and difficulties accessing hygiene.

**Table 1:** Demographic features and comorbidities of 80 patients with crusted scabies, 2016–2018.

Patient characteristics	Number of patients/ number overall
*Age* * Median* * Minimum* * Maximum*	47 years 3 months 87 years
*Female*	47 (59%)
*Indigenous Australian*	76 (95%)
*Urban location*	20 (25%)
*Rural location*	3 (4%)
*Remote location*	57 (71%)
*Diabetes*	40 (50%)
*Kidney failure (dialysis)*	18 (23%)
*Chronic lung disease*	16 (20%)
*Kidney transplant*	2 (3%)
*Chronic liver disease*	7 (9%)
*HIV*	0 (0)
*HTLV-1*	4 (5%)
*Other immunosuppression*	3 (4%)
*Hazardous alcohol use* ^a^	30/76 (39%)
*No comorbidities*^*b*^	14 (18%)
*Documented previous episode of crusted scabies*	25 (31%)

HIV human immunodeficiency virus, HTLV Human T-lymphotropic virus

^a^Alcohol history not available for 4 patients

^b^ This does not include cardiac and other non-immunosuppressive comorbidities

Diabetes was the commonest comorbidity, being present in half of patients, while 23% were patients on renal dialysis. While those 14 with no documented comorbidities were generally otherwise healthy, without evident neurological or cognitive impairment, a classification of ‘no comorbidity’ does not preclude the presence of a non-immunosuppressive background medical history such as heart disease. Of note, the absence of evident comorbidity was more common in those with Grade 3 crusted scabies (9/21, 43%) than in those with Grade 1 or 2 crusted scabies (5/59, 8%) (Chi^2^, p = 0·001).

Hospital admission characteristics are summarised in Table 2. While 12 episodes (13%) required management in the intensive care unit, only 2 of these were attributable to sepsis with bacteremia secondary to scabies. The remaining had other complex medical issues including bowel perforation, encephalopathy and respiratory failure. Four episodes were not admitted to hospital; 2 were managed in a nursing home and 2 in a primary care clinic. Blood cultures were positive in 11 episodes (12%), 6 with methicillin sensitive *Staphylococcus aureus* (MSSA), 4 with methicillin resistant *Staphylococcus aureus* (MRSA) and 1 with *Streptococcus pyogenes* alone. In addition, 4 of those with MSSA also had other organisms cultured (*Pseudomonas aeruginosa*, *Serratia marcescans*, *Acinetobacter baumannii* and *Streptococcus pyogenes*). Bacteremia was present in 5/21 (24%) of those with Grade 3 crusted scabies and 6/71 (8%) of those with Grade 1 or 2 crusted scabies (Chi^2^, p = 0·06).

**Table 2:** Characteristics of hospital admission.

	Overall (n = 92)	Grade 1 (n = 35)	Grade 2 (n = 36)	Grade 3 (n = 21)
LOS (days) Median Minimum Maximum	14 1 151	9 1 151	14 1 150	21 5 45
Scabies as primary reason for admission, n (%)	54 (59%)	14 (40%)	26 (72%)	18 (86%)
DAMA, n (%)	22 (24%)	9 (26%)	8 (22%)	5 (24%)
ICU admission, n (%)	12 (13%)	6 (17%)	5 (14%)	1 (5%)
Secondary SSTI, n (%)	20 (22%)	2 (6%)	11 (31%)	7 (33%)
Bacteremia, n (%)	11 (12%)	2 (6%)	4 (11%)	5 (24%)

LOS length of stay, DAMA self-discharge against medical advice, ICU intensive care unit, SSTI skin and soft tissue infection

Skin swabs were taken in 30 episodes with results summarised in Table 3. Only 1 swab had no growth. The hematological and immunological markers are summarised in Table 4. The neutrophil count was available in 88 episodes and among these, 3 were neutropenic and 37 were neutrophilic. Lymphopenia was present in 49/88 (56%) and eosinophilia in 53/88 (60%), with 33/88 (38%) having an eosinophil count over 1.0 x10^6^mmol/L. The IgE was elevated in 33 of 35 samples tested, with a median value of 1694 kU/L (normal value <26 kU/L).

**Table 3:** Skin swab isolates in episodes with crusted scabies.

	Overall (n = 30)	Grade 1 (n = 7)	Grade 2 (n = 15)	Grade 3 (n = 8)
MSSA^a^	23 (77%)	5 (71%)	11 (73%)	7 (88%)
MRSA^b^	1 (3%)	0 (0%)	1 (7%)	0 (0%)
Strep A^c^	14 (47%)	3 (43%)	8 (53%)	3 (38%)
Other gram-positive organism	3 (10%)	0 (0%)	3 (20%)	0 (0%)
Gram-negative organism	4 (13%)	0 (0%)	3 (20%)	1 (13%)

^a^ Methicillin sensitive *Staphylococcus aureus*

^b^ Methicillin resistant *Staphylococcus aureus*

^c^ Group A streptococcus, *Streptococcus pyogenes*

**Table 4:** Number of episodes with laboratory values outside the reference range.

	*Reference range*	Total Proportion Abnormal	*Range*	Grade 1 Proportion Abnormal	Grade 2 Proportion Abnormal	Grade 3 Proportion Abnormal
*Neutrophils*	2–7·5 x10^6^mmol/L	40/88 (45%) 3 low, 37 high	1·7–23·7	17/33 (52%)	17/35 (49%)	6/20 (30%)
*Lymphocytes*	1·5–4·0 x10^6^mmol/L	49/88 low (56%)	0–7·9	22/33 (67%)	21/35 (60%)	6/20 (30%)
*Eosinophils*	0·0–0·4 x10^6^mmol/L	53/88 high (60%)	0–6·2	15/33 (45%)	23/35 (66%)	15/20 (75%)
*C3*	0·86–1·84 g/L	13/45 low (29%)	0·38–1·38	3/16 (19%)	7/35 (20%)	3/10 (30%)
*C4*	0·20–0·59 g/L	17/45 low (38%)	0–1	7/16 (44%)	5/19 (26%)	5/10 (50%)
*IgE*	<26 kU/L	33/35 (94%) high	8–37780	9/9 (100%)	15/17 (88%)	9/9 (100%)

C3, Complement 3; C4, Complement 4; IgE, Immunoglobulin E

The sites of scabies on the body were available for 77 of 92 episodes (84%). The feet were most commonly involved, followed by the rest of the lower limbs. The hands and buttock were also commonly involved. The trunk, groin and axillae were more likely to be involved in Grade 2 and 3 crusted scabies. Crusted scalp lesions were present in 5/77 (6%) episodes where documented.

Treatment characteristics are summarised in Table 5. All but 1 crusted scabies patient received oral ivermectin and all but 1 episode of crusted scabies was managed with the addition of topical scabicides, with topical therapy declined in this case because of skin irritation. Thirteen doses of ivermectin were provided in 1 case to a patient who continued to self-discharge against medical advice over a 5-month period and did not have a negative scraping until day 103 after initial presentation. Fifty-one episodes completed therapy as hospital inpatients and 22 had planned discharges with support to complete therapy as an outpatient after commencing therapy in hospital. The majority of the remaining 19 episodes were self-discharges without confirmed completion of treatment.

**Table 5:** Treatment characteristics for crusted scabies.

	Overall (n = 92)	Grade 1 (n = 35)^a^	Grade 2 (n = 36)^a^	Grade 3 (n = 21)^a^
Ivermectin therapy (doses) Median Minimum Maximum	5 0 13	3 1 7	5 0 7	7 2 13
Completion of therapy, n In hospital As an outpatient^b^ Not completed^c^	51 (55%) 22 (24%) 19 (21%)	24 (69%) 4 (11%) 7 (20%)	19 (53%) 9 (25%) 8 (22%)	8 (38%) 9 (43%) 4 (19%)
Negative skin scraping^d^, n	65 (71%)	21 (60%)	26 (72%)	18 (86%)
Day of negative scraping (day)^e^ Median Minimum Maximum	12 1 103	11 3 30	9.5 1 23	17·5 6 103

^a^ The recommended dosing of ivermectin is: Three doses for Grade 1 crusted scabies, five doses for Grade 2 crusted scabies and seven doses for Grade 3 crusted scabies.

^b^ In some cases, there was directly observed therapy however completion cannot be confirmed for all. Of those who completed therapy as an outpatient, 50% of Grade 1 cases, 89% of Grade 2 cases and 100% of Grade 3 cases had a documented negative skin scraping for scabies mites before discharge.

^c^ Most cases who failed to complete therapy also self-discharged against medical advice. Only 4 (21%) of these cases had documented negative skin scrapings.

^d^ The number of episodes with documented negative skin scraping for scabies mites before discharge.

^e^ This does not necessarily indicate day of infection clearance, as skin scrapings were not routinely performed after admission, nor at specified intervals, with many patients having no residual skin hyperkeratosis for scraping.

In addition to the 10 patients who had recurrences during the 2-year study period, a further 8 had a first recurrence in the 22-month follow up period, making a total 18 (22.5%) of the 80 patients who had 1 or more repeat presentations with confirmed recurrent crusted scabies. Age at recurrence ranged from 5–79 years. They were all Indigenous, 9 were diabetic, 1 was on dialysis, 5 had hazardous alcohol intake and 1 had HTLV-1 infection. Overall, 8 patients had a single recurrence, 6 had two, 3 had three and 1 had four recurrences. The time interval between episodes is shown in Table 6. Of the total of 33 recurrences, 1 was within 1 month of the preceding presentation, 1 between 1 and 3 months, 2 between 3 and 6 months, 10 more than 6 months and 19 more than 12 months after the previous presentation.

**Table 6:** Time interval between presentations with crusted scabies in 18 patients with recurrent disease.

Episode	Number	Minimum time (months)	Maximum time (months)	Median (months)
First recurrence	18	1	30	12
Second recurrence	10	6	24	12
Third recurrence	4	2	15	12
Fourth recurrence	1	10	10	10

Overall 13 (16%) of the 80 patients died, 7 during the 2 years of the study and 6 during the 22-month follow up period. Three died during their admission with crusted scabies, all having multiple severe comorbidities. Of these, one aged 55 years had MSSA bacteremia and died in the intensive care unit 8 days after admission. The other 2 aged 53 and 79 years, were provided palliative care for their comorbidities and died at 9 and 10 days after admission. One patient with severe liver disease, aged 49 years, died 7 months after admission and treatment for crusted scabies. This patient did not have recurrent crusted scabies but died under palliative care with MSSA bacteremia secondary to severe impetigo. The other 9 deaths were all attributed to severe comorbidities and occurred subsequent to discharge following the treatment of crusted scabies. None had recurrent crusted scabies and 5 were under active palliative care at the time of death.

## Discussion

The current study summarises cases of crusted scabies in the NT in the 2 years since the disease was made notifiable in March 2016. Ninety-two episodes were reported in 80 patients, with the majority being Grade 1 and 2 crusted scabies. More cases were documented in females and 14/80 (18%) individuals had no identified risk factor for crusted scabies. Treatment was completed as per protocol in up to 80% of cases but there were still 33 subsequent recurrent episodes documented amongst 18 of the patients.

Very few studies have documented epidemiology, comorbidities and outcomes for crusted scabies beyond individual case reports. Two other studies have been conducted in Darwin, with findings similar to the current study; the median age in years was in the mid-40s, 97–100% were Indigenous and 20–26% had immunosuppressive comorbidities [6,10]. In one of these prior studies, 42% had no identified comorbidities [10], in comparison to 16% in the current study. The lower proportion seen in the current study, which was undertaken in the same population group as the earlier studies, may simply reflect better ascertainment of comorbidities. Diabetes was the most frequently noted comorbidity (50% of patients) in this cohort, followed by hazardous alcohol use and end-stage kidney failure requiring dialysis. In other studies globally, extremes of age, low socio-economic status, illiteracy, poor hygiene, sharing clothes [11,12] and dementia [2] have been linked to crusted scabies, usually with an older adult population.

In the current study 23% of patients were on renal dialysis, with none of these patients having Grade 3 crusted scabies. While this likely reflects the earlier diagnosis in patients who are under regular nursing and medical care, it also indicates transmission of scabies in this population, supporting a recommendation for regular whole-body skin checks in dialysis patients in areas endemic for scabies and impetigo. HIV and HTLV-1 infection are recognised risk factors for crusted scabies, with the first description linking crusted scabies to patients infected with HTLV-1 being in Indigenous Australians from Central Australia [13]. While skin manifestations of HTLV-1 have been increasingly recognised globally [14] and HTLV-1 is common in Central Australia [15], only 4 of the patients with crusted scabies over the 2 year period were in patients documented to be infected with HTLV-1 and no cases occurred in those known to be HIV positive (numbers living with HIV over the 2 years estimated in the NT at 200–250).

There is evidence that progression to crusted scabies is an immune mediated process [16]. IgE, mast cells and eosinophils are implicated in the pathogenesis of crusted scabies [17]. Elevated IgE [10,16] and eosinophilia [10] have been commonly noted in the current and previous studies [10,16]. Nevertheless, while very elevated IgE was almost universal in the current study, eosinophil count was normal in 40% of crusted scabies episodes. Complement levels were low in just under a third of the cases (where it was tested), with no clear difference between the different grades of scabies.

Ten episodes in the current study had a staphylococcal bacteremia, of which 4 were MRSA staphylococcal infections. *S*. *aureus* was also the most common organism isolated from skin swabs but only 1 of 24 *S*. *aureus* skin isolates was MRSA. Antibiotic therapy was considered necessary in most of these cases. The range of bacteremic and skin pathogens in this cohort included gram-negative bacteria, consistent with previous studies and supporting the recommendation in the NT crusted scabies guidelines for coverage of gram-negative as well as gram-positive bacteria when a patient has sepsis on presentation [8], with piperacillin-tazobactam being commonly used in such circumstances.

In contrast to historical NT data [10,18], among the 80 patients there was only 1 death with sepsis following admission with crusted scabies and intensive care was not commonly required. This likely reflects the aggressive guideline-based management of patients with crusted scabies in the NT; hospital admission, blood cultures and early antibiotic therapy and multiple doses of ivermectin together with supervised topical therapy and skin care with keratolytic cream and bathing [6–8]. Nevertheless, short term mortality in the patient cohort after discharge was high and directly attributable to the multiple and often end-stage comorbidities.

Outbreaks of scabies have been frequently described from aged care and other institutional facilities and these outbreaks may start with an index case of previously unrecognised crusted scabies [2]. A review found crusted scabies as the source in 83% of institutional scabies outbreaks [4]. When such scabies outbreaks occur, attack rates in facilities range from 5–38% [4,19]. Furthermore, healthcare workers in the facilities are also at risk [20], with scabies rates in staff documented up to 30% [4]. Cases have also been reported in family members of these health care workers [20]. The average number of secondary cases from reported scabies institutional outbreaks was 26, with these secondary cases commonly presenting up to 3 months after the index case [4]. Two of the 3 non-Indigenous cases in the cohort reported in our study resided in nursing homes.

Treatment and prevention of further scabies transmission can be effective with adequately planned and executed control and prevention strategies [2,4,20,21] even when immunocompromised hosts are involved [21]. While historically topical therapy alone has been successful for treating crusted scabies [22], oral ivermectin together with topical scabicide and topical keratolytic therapy is now established as the therapy of choice [7]. Nevertheless the number of doses of ivermectin required and the timing of these and of the topical therapy has evolved empirically from the NT experience in managing crusted scabies over the last 2 decades and has not been subjected to any formal controlled studies [6,7,10,21,23–25].

The current study also demonstrates that there is significant burden of crusted scabies on hospital bed days. The median time spent in hospital was 14 days for a disease that is otherwise preventable. Furthermore, cases of crusted scabies require stay in isolation rooms with staff using personalised protective equipment, representing a significant financial burden on the hospital system and adverse effects of isolation for the patient.

Of note, those with Grade 3 crusted scabies had significantly fewer comorbidities than those with less severe disease. It remains possible that a minority of the population has a genetic predisposition that confers inability to contain scabies infection, with consequent unfettered hyperinfestation which manifests as crusted scabies if early diagnosis and therapy are not available or implemented [10,16,17,23–25]. Whether this this possibility of a specific genetic predisposition to crusted scabies explains those cases seen in overtly normal hosts with no documented comorbidities requires further study.

An important consideration for those with repeat episodes of crusted scabies is whether the recurrent episode is recrudescence of inadequately treated initial infection, or a new scabies infection after the susceptible host returns to a scabies-endemic community after successful parasite clearance. Earlier genotyping studies of *S*. *scabiei* from recurrent crusted scabies provided some insight and supported the incremental ivermectin dosing based on severity grading for mite eradication in severe cases [6,7,18]. Following successful therapy with restoration of healthy skin it has been observed that clinically evident recurrent crusted scabies after return to a scabies-endemic community where reinfection occurs takes between 3 and 12 months to develop [18]. In our cohort only 2 of the 33 episodes of recurrent crusted scabies were diagnosed within 3 months of the prior episode and these likely represent recrudescence of inadequately treated infection. The remaining 31 are likely to be new infections following return of the cured patient to their scabies-endemic community.

The public health implications of this study relate to the recognition that undiagnosed crusted scabies patients can be index cases in outbreaks of scabies in residential facilities such as nursing homes and can undermine the success of scabies mass drug administration programs. The important role of undiagnosed and untreated or under-treated patients with crusted scabies in maintaining scabies transmission cycles in remote NT communities is supported by the numbers of confirmed cases found over the 2 years of this study. The ‘One Disease’ program in the NT has been addressing this concern with an emphasis on improved recognition of crusted scabies by health staff and community members [26]. An additional element of the program is having those treated for crusted scabies return from hospital to a ‘scabies free zone’, through treatment of community contacts and funding of household and community support initiatives (https://onedisease.org), thereby preventing reinfection of those recognised as susceptible to developing crusted scabies and being potential core transmitters of scabies. Success of this program will be judged by documenting a future substantial decrease in the number of recurrent episodes of crusted scabies in comparison to those seen over the 2 years of this study and the subsequent 22 months follow up. The elimination of repeated crusted scabies cases will not only decrease overall scabies transmission but will also make future mass drug administration (MDA) programs in the NT far more likely to succeed.

Recent studies from the Pacific have shown how successful a MDA can be in decreasing scabies rates in populations [27]. However, the success of an MDA can be limited in highly mobile populations, where reintroduction of scabies into the community occurs from newcomers or returned community members who have not been treated. Such has been the situation with prior scabies MDAs in the NT [28]. The diagnosis and treatment of crusted scabies in the NT and prevention of recurrent cases is a critical consideration prior to any future MDAs. Additional important factors include wider targeting of any MDA to concurrently cover multiple communities in a region and the potential future use of oral moxidectin as a possible superior therapy than ivermectin for scabies and scabies MDAs [29,30].

The limitations to this study include that while the cases were identified prospectively with core data collected, the expanded data were collected retrospectively. Therefore, the estimate in this study that 16% of patients with crusted scabies had no comorbidity may still be an overestimate. In addition, while the guidelines recommend that all patients with crusted scabies are tested for HIV and HTLV-1, we were unable to verify this was done for all the cohort. Specific community location and housing data and crowding were not assessed. Such data would provide potential insights into scabies transmission cycles and the epidemiology of recurrent crusted scabies. In addition, there may have been cases of crusted scabies in remote regions of the NT which were not recognised or were not notified because skin scrapings for diagnosis were not performed. The assessment that all but 2 of the 33 recurrences of crusted scabies were likely to be new infections following return of the cured patient to their scabies-endemic community, rather than recrudescence of inadequately treated disease, is limited by the issues of patient self-discharge, some loss to follow up and skin scrapings for clearance not being universal.

In conclusion, a mandatory notification system with robust diagnostic criteria has provided important insights into crusted scabies on a population level. The current study highlights the burden of crusted scabies in the Indigenous population in central and northern Australia. Despite scabies being a preventable disease, a large number of crusted scabies cases were identified within a 2-year period. This justifies concern that crusted scabies remains a disease of significant morbidity to the individual and their well-being and to their families and communities as they can remain core transmitters in the ongoing scabies epidemics in remote Indigenous communities. It is also critical to note that the primordial factors underlying the current high rates of scabies in remote Indigenous communities are centred around continuing socioeconomic disadvantage.
